# Harnessing the immune response to target tumors

**DOI:** 10.12688/f1000research.10795.1

**Published:** 2017-05-18

**Authors:** Luisa Manning, John Nemunaitis

**Affiliations:** 1 Gradalis, Inc., Dallas, TX, USA; 2 Mary Crowley Cancer Research Center, Dallas, TX, USA

**Keywords:** targeted immune therapy, anticancer response, checkpoint inhibitors, adoptive T-cell therapy, CAR-T cell therapy

## Abstract

Development of “immune-based targeted therapy” in oncology has limited experience with signal pathway modulation. However, as we have become better versed in understanding immune function related to anticancer response, “hints” of specific targets associated with sensitivity and resistance have been identified with targeted immune therapy. This brief review summarizes the relationship of several targeted immune therapeutics and activity associated clinical responsiveness.

## Introduction

Although the immune system distinguishes self versus non-self antigens with the intent to eliminate cells expressing non-self antigens, cancer cells have developed mechanisms to escape or suppress the “non-self attack”, thereby enabling tumor proliferation and progression. Increasing numbers of innovative immunotherapies are being developed that address immune modulation of non-self targets to reverse cancer defenses.

## Checkpoint inhibitors

Several targets have been associated with evidence of clinical benefit, resulting in a broad spectrum of investigational and approved immunotherapies. These include cellular therapies (adoptive T-cell and dendritic cell therapy, cytokine-induced killer cells, tumor vaccines, and autologous tumor cell therapy) and checkpoint inhibitors (PD-1/L-1 and CTLA-4 inhibitors). Several checkpoint inhibitors have shown superior clinical benefit over standard of care ^1– 3^; however, a high percentage of patients do not show durable response rates in monotherapies or combination therapies with checkpoint blockade. Factors like tumor-infiltrating lymphocyte (TIL) infiltration and PD-L1 expression levels are associated with response ^4^. In addition, tumor mutation burden (TMB) appears to be a prognostic marker for immune response ^5^. During cancer cell proliferation, somatic mutations increase the expression of a variety of tumor-associated antigens and neoantigens. Studies show that high TMB correlates with the amount of immunogenic neoantigens ( *P* <0.0001), presented by major histocompatibility complex (MHC) molecules to immune effector cells, inducing higher durable immune responses (overall response rate of 63% versus 0%; *P* = 0.03) and progression-free survival prolongation (14.5 versus 3.7 months; *P* = 0.01) than in tumor types with lower mutation burden ^6^. Regardless of histology type, tumors with a mean mutational load of more than 10 somatic mutations per megabase of coding DNA appear more likely to be immunogenic to effector T cells eliciting antitumor immunity ^7, 8^. Other cancer types, such as colorectal cancer, and interestingly subgroups with a high number of somatic mutations and potential mutation-associated neoepitopes appear to correlate with higher responses to checkpoint inhibitors in mismatch repair-deficient tumors ^9^. Recent studies show that the overall response rate to PD-1/L-1 therapies in high TMB tumor types has been durable for years with delayed relapse or disease progression ^10^. On the other hand, signal pathways, such as those associated with interferon receptor expression related to loss of JAK 1 or JAK 2 function, result in unresponsiveness to interferon gamma, a common antiproliferative cytokine associated with oncolytic activity. This effect has been well demonstrated in a subset of PD-1/L1-refractory patients. Zaretsky *et al*. ^10^ identified inactivating mutations in JAK 1 and 2 that silence the CD8 T cell-induced interferon gamma signaling cascade, an adaptive antitumor response. Another mechanism such as beta 2-microglobulin inactivation results in loss of MHC1 expression. Moreover, mutations of death receptors—like Fas or tumor necrosis factor-related apoptosis-inducing ligand—are associated with insensitivities against granzymes or perforin or both, which also play major roles in immune escape and resistance.

Bu *et al.* ^11^ discussed PD-1 resistances and highlighted a pattern of upregulated genes first observed in patients with PD-1-resistant melanoma ^12^, termed innate PD-1 resistance effect. The analysis of somatic mutanomes and transcriptomes of pretreatment melanoma biopsies included the comparison of differentiated gene expression in PD-1 responders versus non-responders. Higher expressed genes in checkpoint non-responding tumors included mesenchymal transition genes ( *AXL*, *ROR2*, *WNT5A*, *LOXL2*, *TWIST2*, *TAGLN*, and *FAP*), immunosuppressive genes ( *IL10*, *VEGFA*, and *VEGFC*), and monocyte and macrophage chemotactic genes ( *CCL2*, *CCL7*, *CCL8*, and *CCL13*) ^12^, while immune responsive tumors also contained transforming growth factor beta (TGFβ) signaling defects.

To address and overcome resistant mechanisms, ongoing studies are extensively investigating combination approaches (that is, with checkpoint inhibitors). For example, experiments of targeted inhibition of mitogen-activated protein kinase show synergy with PD-1/L1 pathway inhibition and increases in CD8 T-cell number within the tumor environment in association with increased tumor response ^13^.

## Adoptive T-cell therapies

Adoptive dendritic cell or T-cell therapies show clinically meaningful value in hematologic malignancies, and a small number of case reports support efficacy in solid tumors with demonstration of durable clinical responses ^14– 16^. For example, 20 to 25% of patients with metastatic melanoma showed durable responses to expanded TIL therapies ^14, 17^. This is most likely related to neoantigen signal identification. A remarkable case of response of adoptive T-cell therapy to a common neoantigen target was recently demonstrated to *KRAS* G12D mutation ^16^ and other, lesser-known mutations ^14, 15^. However, currently, the majority of cancer vaccines and adoptive T-cell approaches fall short of significant efficacy targeting pre-selected MHC-dependent (genetically modified T cells) or independent—chimeric antigen receptor-T (CAR-T)—antigens showing limited activity in solid tumors, possibly related to the lack of knowledge of relevant neoantigens ( Table 1). While CD19-targeting CAR-T cell therapies have demonstrated curative events in B-cell malignancies ^18, 19^, efficacy in solid tumors appears to be limited by heterogeneity, lack of relevant tumor-specific or -associated antigens and low immunogenicity ^20^, in balance with other immunosuppressive pathways not addressed within the tumor microenvironment.

**Table 1.** Cellular immunotherapies.

Target	Tumor type(s)	Reference/Clinical trial(s)
Chimeric antigen receptor–T cell therapies/targets		
CD19	B-cell malignancies	18, 19; NCT02975687; NCT02842138; NCT02813837
Mesothelin	Mesothelioma, lung cancer, breast cancer	36, 37; NCT02930993; NCT02706782
L1-CAM	Metastatic neuroblastoma	38; NCT02311621
GD2	Neuroblastoma	25, 39; NCT02107963; NCT02919046
Lewis Y	Myeloid malignancies	24, 40; NCT01716364
EGFRvIII	Brain tumor	NCT01454596
HER2	Colon cancer, HER2-positive lung cancer, malignant glioma, Her2-positive sarcoma	26, 41; NCT00889954; NCT00902044; NCT01109095
CD20	Follicular and mantel cell lymphoma	42, 43; NCT00621452
CEA	Stomach cancer, metastatic adenocarcinoma, breast cancer	44; NCT00673829; NCT00673322
MUC-16/IL-12	Ovarian cancer	45; NCT02498912
WT1	Acute myeloid leukemia, NSCLC, breast, pancreatic, ovarian, colorectal cancer, mesothelioma	27, 28; NCT02408016
CAIX	Renal cell carcinoma	46, 47
FAP	Malignant pleural mesothelioma	48
PSMA	Prostate cancer	NCT00664196; NCT01140373
Kappa light chain (klC)	B-cell lymphoma, chronic lymphocytic leukemia, multiple myeloma	NCT00881920
CD30	Hodgkin’s lymphoma, non-Hodgkin’s lymphomas	NCT01316146
HLA-A1/MAGE1	Melanoma	49, 50
HLA-A2/NY-ESO-1	Sarcoma, melanoma	51
MUC1	Ovarian, breast, pancreas, colorectal, malignant glioma, NSCLC, hepatocellular	52; NCT02587689; NCT02617134; NCT02839954
VEGFR-2	Solid tumors	53, 54; NCT01218867
Adoptive cell therapies		
Autologous tumor-infiltrating lymphocyte therapy and IL-2	Metastatic melanoma	14
Dendritic cell vaccine and cytokine- induced killer cell therapy	Hepatobiliary, pancreatic cancer	55
Adoptive T-cell transfer	Metastatic melanoma	15
Dendritic cell-derived exosomes (Dex)	NSCLC, melanoma, colorectal cancer	56– 59
Adoptive CD8 ^+^ T cells	KRAS, G12D, colorectal	16
Autologous tumor cell therapy		
Vigil EATC	Ovarian cancer, Ewing’s sarcoma, NSCLC, melanoma, triple-negative breast cancer, solid tumors	32– 35

IL, interleukin; NSCLC, non-small cell lung cancer.

Adoptive T-cell therapy “exhaustion” may also be influenced by upregulation of pathways such as PD-L1 expression on tumor cells. Strategies to convert the negative signal of PD-L1 to co-stimulatory receptors by PD1:28 chimer alteration showed encouraging results in activation of CD8 effector T cells ^21^. Tran *et al*. identified CD8 T-cell responses against mutant *KRAS* G12D and HLA-C*08:02 in a patient with colorectal cancer, receiving a single-dose infusion of 1.48 × 10 ^11^ TILs (approximately 75% CD8 T cells) with durable regression of lung metastases with disease progression 9 months after treatment ^16^.

## Tumor signaling/microenvironment modulation

Overcoming tumor-induced immunosuppression can also involve tumor signal modulation and microenvironment influence. Altered expressions of survival genes (Bcl-xL), increasing the expression of dominant negative TGFβ receptors to overcome inhibitory effects ^22^, regulatory T suppression, indoleamine 2,3-dioxygenase downregulation, and other signaling microenvironment therapeutics, such as WNT/β-catenin signaling pathway ^23^, are being tested to address benefit opportunity.

## CAR-T: selective antigen targets

Targeting driver mutations or their *de novo* neoepitopes are very attractive and appear to be very promising in effective anticancer therapies. There are several cancer-associated or -specific antigen-loaded CAR-T cell therapies, selected by different algorithms, in clinical trials to investigate further efficacy in solid tumors ( Table 1). *In vivo* activity of gene-modified T cells was demonstrated in the delayed growth and prolonged survival of Lewis Y antigen CAR-T cell therapy in lymphoma with the report of two cases with stable disease ^24^. Louis *et al*. reported other responses, including three complete responses in patients with neuroblastoma treated with specific CAR-T cells targeting GD2 ganglioside ^25^. HER2-positive colon, lung cancer, and sarcomas are also under investigational therapy with HER2 CAR-T therapy, showing promising results with stable diseases for 12 weeks up to 14 months but no partial or complete responses in HER2-positive sarcomas ^26^. Other targets—that is, carcinoembryonic antigen (CEA) in colon cancer and WT1 in mesothelioma and ovarian cancer ^27, 28^—are being studied as well. Among the most challenging aspects of adoptive cell therapies and CAR-T engineering are the identification and use of antigens for focused immune effector cell activation to cancer targets only. Despite the large number of investigated tumor antigens with limited encouraging results, high rates of undesirable off-tumor effects, such as cytokine-release syndrome (CRS) or other immune-related adverse events, are widely seen in CAR-T cell therapies. Thus, new approaches with implications for suicide genes like inducible caspase9 or herpes simplex thymidine kinase are under investigation to enhance the safety of T-cell therapies ^29, 30^ along with novel regimens to directly address CRS (that is, interleukin-6 inhibitor) ^31^.

Interestingly, one investigational personalized cellular immunotherapy product with a mechanism directly associated with autologous DNA engineered tumor cells called Vigil ^32– 35^ shows evidence of enhanced tumor-specific antigen targeting via effector T-cell activation in correlation with clinical benefit in solid tumors. Autologous tumor cells include the full patient- and tumor-specific antigen repertoire. This is a unique aspect of the Vigil therapy.

## Conclusions

The future is bright for combination immunotherapy, particularly as exact targets are identified with the tumor microenvironment, thereby enabling access to tumor “non-self” neoantigens.

## References

[1] Rizvi NA Mazières J Planchard D: Activity and safety of nivolumab, an anti-PD-1 immune checkpoint inhibitor, for patients with advanced, refractory squamous non-small-cell lung cancer (CheckMate 063): a phase 2, single-arm trial. *Lancet Oncol.* 2015;16(3):257–65. 10.1016/S1470-2045(15)70054-9 25704439 PMC5726228

[2] Rosenberg JE Hoffman-Censits J Powles T: Atezolizumab in patients with locally advanced and metastatic urothelial carcinoma who have progressed following treatment with platinum-based chemotherapy: a single-arm, multicentre, phase 2 trial. *Lancet.* 2016;387(10031):1909–20. 10.1016/S0140-6736(16)00561-4 26952546 PMC5480242

[3] Hamanishi J Mandai M Ikeda T: Safety and Antitumor Activity of Anti-PD-1 Antibody, Nivolumab, in Patients With Platinum-Resistant Ovarian Cancer. *J Clin Oncol.* 2015;33(34):4015–22. 10.1200/JCO.2015.62.3397 26351349

[4] Ameratunga M Asadi K Lin X: PD-L1 and Tumor Infiltrating Lymphocytes as Prognostic Markers in Resected NSCLC. *PLoS One.* 2016;11(4):e0153954. 10.1371/journal.pone.0153954 27104612 PMC4841565

[5] Burke JM Lamm DL Meng MV: A first in human phase 1 study of CG0070, a GM-CSF expressing oncolytic adenovirus, for the treatment of nonmuscle invasive bladder cancer. *J Urol.* 2012;188(6):2391–7. 10.1016/j.juro.2012.07.097 23088985

[6] Rizvi NA Hellmann MD Snyder A: Cancer immunology. Mutational landscape determines sensitivity to PD-1 blockade in non-small cell lung cancer. *Science.* 2015;348(6230):124–8. 10.1126/science.aaa1348 25765070 PMC4993154

[7] Alexandrov LB Nik-Zainal S Wedge DC: Signatures of mutational processes in human cancer. *Nature.* 2013;500(7463):415–21. 10.1038/nature12477 23945592 PMC3776390

[8] Schumacher TN Schreiber RD: Neoantigens in cancer immunotherapy. *Science.* 2015;348(6230):69–74. 10.1126/science.aaa4971 25838375

[9] Le DT Uram JN Wang H: PD-1 Blockade in Tumors with Mismatch-Repair Deficiency. *N Engl J Med.* 2015;372(26):2509–20. 10.1056/NEJMoa1500596 26028255 PMC4481136

[10] Zaretsky JM Garcia-Diaz A Shin DS: Mutations Associated with Acquired Resistance to PD-1 Blockade in Melanoma. *N Engl J Med.* 2016;375(9):819–29. 10.1056/NEJMoa1604958 27433843 PMC5007206

[11] Bu X Mahoney KM Freeman GJ: Learning from PD-1 Resistance: New Combination Strategies. *Trends Mol Med.* 2016;22(6):448–51. 10.1016/j.molmed.2016.04.008 27174038 PMC6833952

[12] Hugo W Zaretsky JM Sun L: Genomic and Transcriptomic Features of Response to Anti-PD-1 Therapy in Metastatic Melanoma. *Cell.* 2016;165(1):35–44. 10.1016/j.cell.2016.02.065 26997480 PMC4808437

[13] Ebert PJ Cheung J Yang Y: MAP Kinase Inhibition Promotes T Cell and Anti-tumor Activity in Combination with PD-L1 Checkpoint Blockade. *Immunity.* 2016;44(3):609–21. 10.1016/j.immuni.2016.01.024 26944201

[14] Rosenberg SA Yang JC Sherry RM: Durable complete responses in heavily pretreated patients with metastatic melanoma using T-cell transfer immunotherapy. *Clin Cancer Res.* 2011;17(13):4550–7. 10.1158/1078-0432.CCR-11-0116 21498393 PMC3131487

[15] Verdegaal EM de Miranda NF Visser M: Neoantigen landscape dynamics during human melanoma-T cell interactions. *Nature.* 2016;536(7614):91–5. 10.1038/nature18945 27350335

[16] Tran E Robbins PF Lu YC: T-Cell Transfer Therapy Targeting Mutant KRAS in Cancer. *N Engl J Med.* 2016;375(23):2255–62. 10.1056/NEJMoa1609279 27959684 PMC5178827

[17] Goff SL Dudley ME Citrin DE: Randomized, Prospective Evaluation Comparing Intensity of Lymphodepletion Before Adoptive Transfer of Tumor-Infiltrating Lymphocytes for Patients With Metastatic Melanoma. *J Clin Oncol.* 2016;34(20):2389–97. 10.1200/JCO.2016.66.7220 27217459 PMC4981979

[18] Park JH Geyer MB Brentjens RJ: CD19-targeted CAR T-cell therapeutics for hematologic malignancies: interpreting clinical outcomes to date. *Blood.* 2016;127(26):3312–20. 10.1182/blood-2016-02-629063 27207800 PMC4929923

[19] Brentjens RJ Rivière I Park JH: Safety and persistence of adoptively transferred autologous CD19-targeted T cells in patients with relapsed or chemotherapy refractory B-cell leukemias. *Blood.* 2011;118(18):4817–28. 10.1182/blood-2011-04-348540 21849486 PMC3208293

[20] Martinet L Garrido I Filleron T: Human solid tumors contain high endothelial venules: association with T- and B-lymphocyte infiltration and favorable prognosis in breast cancer. *Cancer Res.* 2011;71(17):5678–87. 10.1158/0008-5472.CAN-11-0431 21846823

[21] Prosser ME Brown CE Shami AF: Tumor PD-L1 co-stimulates primary human CD8 ^+^ cytotoxic T cells modified to express a PD1:CD28 chimeric receptor. *Mol Immunol.* 2012;51(3–4):263–72. 10.1016/j.molimm.2012.03.023 22503210

[22] Foster AE Dotti G Lu A: Antitumor activity of EBV-specific T lymphocytes transduced with a dominant negative TGF-beta receptor. *J Immunother.* 2008;31(5):500–5. 10.1097/CJI.0b013e318177092b 18463534 PMC2745436

[23] Spranger S Bao R Gajewski TF: Melanoma-intrinsic β-catenin signalling prevents anti-tumour immunity. *Nature.* 2015;523(7559):231–5. 10.1038/nature14404 25970248

[24] Peinert S Prince HM Guru PM: Gene-modified T cells as immunotherapy for multiple myeloma and acute myeloid leukemia expressing the Lewis Y antigen. *Gene Ther.* 2010;17(5):678–86. 10.1038/gt.2010.21 20200563

[25] Louis CU Savoldo B Dotti G: Antitumor activity and long-term fate of chimeric antigen receptor-positive T cells in patients with neuroblastoma. *Blood.* 2011;118(23):6050–6. 10.1182/blood-2011-05-354449 21984804 PMC3234664

[26] Ahmed N Brawley VS Hegde M: Human Epidermal Growth Factor Receptor 2 (HER2) -Specific Chimeric Antigen Receptor-Modified T Cells for the Immunotherapy of HER2-Positive Sarcoma. *J Clin Oncol.* 2015;33(15):1688–96. 10.1200/JCO.2014.58.0225 25800760 PMC4429176

[27] Krug LM Dao T Brown AB: WT1 peptide vaccinations induce CD4 and CD8 T cell immune responses in patients with mesothelioma and non-small cell lung cancer. *Cancer Immunol Immunother.* 2010;59(10):1467–79. 10.1007/s00262-010-0871-8 20532500 PMC4004362

[28] Dohi S Ohno S Ohno Y: WT1 peptide vaccine stabilized intractable ovarian cancer patient for one year: a case report. *Anticancer Res.* 2011;31(7):2441–5. 21873157

[29] Gargett T Brown MP: The inducible caspase-9 suicide gene system as a “safety switch” to limit on-target, off-tumor toxicities of chimeric antigen receptor T cells. *Front Pharmacol.* 2014;5:235. 10.3389/fphar.2014.00235 25389405 PMC4211380

[30] Ciceri F Bonini C Stanghellini MT: Infusion of suicide-gene-engineered donor lymphocytes after family haploidentical haemopoietic stem-cell transplantation for leukaemia (the TK007 trial): a non-randomised phase I-II study. *Lancet Oncol.* 2009;10(5):489–500. 10.1016/S1470-2045(09)70074-9 19345145

[31] Lee DW Gardner R Porter DL: Current concepts in the diagnosis and management of cytokine release syndrome. *Blood.* 2014;124(2):188–95. 10.1182/blood-2014-05-552729 24876563 PMC4093680

[32] Senzer N Barve M Nemunaitis J: Long Term Follow Up: Phase I Trial of “bi-shRNA furin/GMCSF DNA/Autologous Tumor Cell” Immunotherapy (FANG™) in Advanced Cancer. *J Vaccines Vaccin.* 2013;4:209 10.4172/2157-7560.1000209

[33] Ghisoli M Barve M Mennel R: Three-year Follow up of GMCSF/bi-shRNA ^furin^ DNA-transfected Autologous Tumor Immunotherapy (Vigil) in Metastatic Advanced Ewing's Sarcoma. *Mol Ther.* 2016;24(8):1478–83. 10.1038/mt.2016.86 27109631 PMC5023386

[34] Oh J Barve M Matthews CM: Phase II study of Vigil® DNA engineered immunotherapy as maintenance in advanced stage ovarian cancer. *Gynecol Oncol.* 2016;143(3):504–10. 10.1016/j.ygyno.2016.09.018 27678295

[35] Barve M Kuhn J Lamont J: Follow-up of bi-shRNA furin / GM-CSF Engineered Autologous Tumor Cell (EATC) Immunotherapy Vigil ^®^ in patients with advanced melanoma. *Biomed Genet Genomics.* 2016;1 10.15761/bgg.1000116

[36] Maus MV Haas AR Beatty GL: T cells expressing chimeric antigen receptors can cause anaphylaxis in humans. *Cancer Immunol Res.* 2013;1(1):26–31. 10.1158/2326-6066.CIR-13-0006 24777247 PMC3888798

[37] Hasegawa K Nakamura T Harvey M: The use of a tropism-modified measles virus in folate receptor-targeted virotherapy of ovarian cancer. *Clin Cancer Res.* 2006;12(20 Pt 1):6170–8. 10.1158/1078-0432.CCR-06-0992 17062694

[38] Park JR Digiusto DL Slovak M: Adoptive transfer of chimeric antigen receptor re-directed cytolytic T lymphocyte clones in patients with neuroblastoma. *Mol Ther.* 2007;15(4):825–33. 10.1038/sj.mt.6300104 17299405

[39] Pule MA Savoldo B Myers GD: Virus-specific T cells engineered to coexpress tumor-specific receptors: persistence and antitumor activity in individuals with neuroblastoma. *Nat Med.* 2008;14(11):1264–70. 10.1038/nm.1882 18978797 PMC2749734

[40] Westwood JA Smyth MJ Teng MW: Adoptive transfer of T cells modified with a humanized chimeric receptor gene inhibits growth of Lewis-Y-expressing tumors in mice. *Proc Natl Acad Sci USA.* 2005;102(52):19051–6. 10.1073/pnas.0504312102 16365285 PMC1323148

[41] Morgan RA Yang JC Kitano M: Case report of a serious adverse event following the administration of T cells transduced with a chimeric antigen receptor recognizing *ERBB2*. *Mol Ther.* 2010;18(4):843–51. 10.1038/mt.2010.24 20179677 PMC2862534

[42] Zhao Y Moon E Carpenito C: Multiple injections of electroporated autologous T cells expressing a chimeric antigen receptor mediate regression of human disseminated tumor. *Cancer Res.* 2010;70(22):9053–61. 10.1158/0008-5472.CAN-10-2880 20926399 PMC2982929

[43] Till BG Jensen MC Wang J: CD20-specific adoptive immunotherapy for lymphoma using a chimeric antigen receptor with both CD28 and 4-1BB domains: pilot clinical trial results. *Blood.* 2012;119(17):3940–50. 10.1182/blood-2011-10-387969 22308288 PMC3350361

[44] Guest RD Kirillova N Mowbray S: Definition and application of good manufacturing process-compliant production of CEA-specific chimeric antigen receptor expressing T-cells for phase I/II clinical trial. *Cancer Immunol Immunother.* 2014;63(2):133–45. 10.1007/s00262-013-1492-9 24190544 PMC11029514

[45] Koneru M Purdon TJ Spriggs D: IL-12 secreting tumor-targeted chimeric antigen receptor T cells eradicate ovarian tumors *in vivo*. *Oncoimmunology.* 2015;4(3):e994446. 10.4161/2162402X.2014.994446 25949921 PMC4404840

[46] Lamers CH Langeveld SC Groot-van Ruijven CM: Gene-modified T cells for adoptive immunotherapy of renal cell cancer maintain transgene-specific immune functions *in vivo.* *Cancer Immunol Immunother.* 2007;56(12):1875–83. 10.1007/s00262-007-0330-3 17479266 PMC11030170

[47] Weijtens ME Willemsen RA van Krimpen BA: Chimeric scFv/gamma receptor-mediated T-cell lysis of tumor cells is coregulated by adhesion and accessory molecules. *Int J Cancer.* 1998;77(2):181–7. 10.1002/(SICI)1097-0215(19980717)77:2<181::AID-IJC2>3.0.CO;2-M 9650549

[48] Petrausch U Schuberth PC Hagedorn C: Re-directed T cells for the treatment of fibroblast activation protein (FAP)-positive malignant pleural mesothelioma (FAPME-1). *BMC Cancer.* 2012;12:615. 10.1186/1471-2407-12-615 23259649 PMC3585825

[49] Willemsen RA Debets R Hart E: A phage display selected fab fragment with MHC class I-restricted specificity for MAGE-A1 allows for retargeting of primary human T lymphocytes. *Gene Ther.* 2001;8(21):1601–8. 10.1038/sj.gt.3301570 11894998

[50] Willemsen RA Ronteltap C Chames P: T cell retargeting with MHC class I-restricted antibodies: the CD28 costimulatory domain enhances antigen-specific cytotoxicity and cytokine production. *J Immunol.* 2005;174(12):7853–8. 10.4049/jimmunol.174.12.7853 15944290

[51] Schuberth PC Jakka G Jensen SM: Effector memory and central memory NY-ESO-1-specific re-directed T cells for treatment of multiple myeloma. *Gene Ther.* 2013;20(4):386–95. 10.1038/gt.2012.48 22739387

[52] Wilkie S Picco G Foster J: Retargeting of human T cells to tumor-associated MUC1: the evolution of a chimeric antigen receptor. *J Immunol.* 2008;180(7):4901–9. 10.4049/jimmunol.180.7.4901 18354214

[53] Chinnasamy D Yu Z Theoret MR: Gene therapy using genetically modified lymphocytes targeting VEGFR-2 inhibits the growth of vascularized syngenic tumors in mice. *J Clin Invest.* 2010;120(11):3953–68. 10.1172/JCI43490 20978347 PMC2964987

[54] Niederman TMJ Ghogawala Z Carter BS: Antitumor activity of cytotoxic T lymphocytes engineered to target vascular endothelial growth factor receptors. *Proc Natl Acad Sci USA.* 2002;99(10):7009–14. 10.1073/pnas.092562399 11997459 PMC124519

[55] Zhang L Zhu W Li J: Clinical outcome of immunotherapy with dendritic cell vaccine and cytokine-induced killer cell therapy in hepatobiliary and pancreatic cancer. *Mol Clin Oncol.* 2016;4(1):129–33. 10.3892/mco.2015.660 26870371 PMC4727101

[56] Escudier B Dorval T Chaput N: Vaccination of metastatic melanoma patients with autologous dendritic cell (DC) derived-exosomes: results of the first phase I clinical trial. *J Transl Med.* 2005;3(1):10. 10.1186/1479-5876-3-10 15740633 PMC554765

[57] Morse MA Garst J Osada T: A phase I study of dexosome immunotherapy in patients with advanced non-small cell lung cancer. *J Transl Med.* 2005;3(1):9. 10.1186/1479-5876-3-9 15723705 PMC551593

[58] Besse B Charrier M Lapierre V: Dendritic cell-derived exosomes as maintenance immunotherapy after first line chemotherapy in NSCLC. *Oncoimmunology.* 2016;5(4):e1071008. 10.1080/2162402X.2015.1071008 27141373 PMC4839329

[59] Dai S Wei D Wu Z: Phase I clinical trial of autologous ascites-derived exosomes combined with GM-CSF for colorectal cancer. *Mol Ther.* 2008;16(4):782–90. 10.1038/mt.2008.1 18362931 PMC7106337

